# Obesity and Risk of Hip Fracture in Adults: A Meta-Analysis of Prospective Cohort Studies

**DOI:** 10.1371/journal.pone.0055077

**Published:** 2013-04-12

**Authors:** Xianye Tang, Gang Liu, Jian Kang, Yang Hou, Fungui Jiang, Wen Yuan, Jiangang Shi

**Affiliations:** 1 Orthopaedic Department, Changzheng Hospital, Second Military Medical University, Shanghai, China; 2 Department of Orthopaedics, People's Hospital of Wujiang City, Wujiang, China; University of Sao Paulo, Brazil

## Abstract

**Background:**

Many observational studies assessed the association between obesity and risk of hip fracture in adults, but reported controversial results. Our goal was to evaluate the association between obesity and risk of hip fracture in adults by conducting a meta-analysis of prospective cohort studies.

**Methods:**

Three databases, PubMed, Embase and Web of Science, were searched through May 2012 to identify eligible cohort studies. Either a fixed- or a random-effects model was used to calculate the pooled relative risk (RR) with its 95% confidence interval (95%CI).

**Results:**

Fifteen prospective cohort studies involving a total 3,126,313 participants were finally included into this meta-analysis. Overall, adults with obesity compared with the normal weight group had a significantly decreased risk of hip fracture (RR: 0.66, 95% CI 0.57 to 0.77, P<0.001). Meta-analyses by the adjusted status of RRs also suggested adults with obesity compared with the reference group had a significantly decreased risk of hip fracture (adjusted RR: 0.48, 95% CI 0.39 to 0.58, P<0.001; unadjusted RR: 0.66, 95% CI 0.56 to 0.78, P<0.001). Subgroup analyses by gender suggested individuals with obesity had a significantly decreased risk for developing hip fracture compared with the reference group in both men (RR 0.54, 95% CI 0.48 to 0.60, P<0.001) and women (RR 0.70, 95% CI 0.58 to 0.84, P<0.001). No evidence of publication bias was observed in this meta-analysis.

**Conclusions:**

This meta-analysis of prospective cohort studies suggests that obesity significantly decreases the risk of hip fracture in adults, and obesity is probably a protective factor of hip fracture in adults.

## Introduction

Osteoporotic fractures are a major and increasing cause of morbidity, and they have caused a serious burden to health services in the world [1], [2]. Hip fracture is a major part of osteoporotic fractures, and is associated with low independence, excess morbidity and high mortality [3], [4]. With the increasing ageing population and the high prevalence of osteoporosis, hip fracture is causing more serious damage to the public health [1], [5]. Thus, to decrease the prevalence of hip fracture, multifaceted interventions for preventing hip fracture are urgently needed [6]–[8]. To make more effective therapeutic or lifestyle interventions, we need have a better knowledge of both the risk factors and the protective factors of hip fracture [9]–[11]. Low body mass index (BMI) is a recognized risk factor for fracture, particularly for the hip fracture [7], [12]. However, whether obesity is protective against fracture has recently been challenged, and the association between obesity and fracture is controversial [13], [14]. As obesity has risen in adults (BMI >30 kg/m2), so has the number of studies examining the association between obesity and fracture risk [7], [14]. Many epidemiologic were published to investigate the association between obesity and risk of hip fracture, but the magnitudes of the association varied among those studies [15]–[29]. Furthermore, whether obesity is an independent protective factor or merely a silent marker of hip fracture remains unclear [13], [14]. An improved understanding of this issue may have important public health and clinical implications given more effective therapeutic or lifestyle interventions can be made [13], [14]. Thus, to evaluate the association between obesity and risk of hip fracture in adults, we performed a meta-analysis of 15 prospective cohort studies [15]–[29]. We performed the meta-analysis by following the PRISMA in the systematic review and meta-analysis [30].

## Methods

### Search strategy

We conducted a literature search in PubMed and Embase databases through May 6, 2012 for relevant studies that tested the association between adult obesity and risk of hip fracture. The following key words were used the literature search: 1) obesity, body mass index, BMI, or overweight; 2) fracture, or fractures; and 3) cohort study, cohort studies, prospective study, or prospective studies or longitudinal study. There was no language restriction in the literature search. In addition, we reviewed the reference lists of retrieved papers and recent reviews for additional studies.

### Study selection

We first performed an initial screening of titles or abstracts. A second screening was based on full-text review. Studies were considered eligible if they met the following criteria: 1) the study design was a prospective cohort study; 2) the exposure of interest was obesity; 3) the outcome of interest was hip fracture; and 4) relative risk (RR) and the corresponding 95% confidence interval (95%CI) (or data to calculate them) were reported.

### Data extraction and quality assessment

Two authors independently conducted the studies selection and data extraction, and any disagreements were resolved by discussion. The key exposure variable was the presence or absence of obesity at baseline. In most studies, people with normal weight served as the reference group. Outcome of interest in this study was hip fracture. Data extraction was then performed using a standardized data-collection form. We extracted any reported RRs, hazard ratios, or incidence density ratios of outcomes for patients with obesity compared with the reference group. We also extracted study characteristics for each trial, and data were recorded as follows: first author's last name, year of publication, country of origin, study period and duration of follow-up, characteristics of study population and age at baseline, number of participants with obesity, number of participants with normal weight, number of hip fracture events, ascertainments of obesity, and statistical adjustments for confounding factors. To clarify that all papers were based on different cohorts, we carefully checked the basic characteristics of included studies including the country of origin, study period, duration of follow-up, characteristics of study population and age at baseline. Quality assessment for cohort studies in this meta-analysis was assessed using the Newcastle Ottawa scale (NOS) as recommended by the Cochrane Non-Randomized Studies Methods Working Group [31]. Given the variability in quality of observational studies found on our initial literature search, we considered studies that met 5 or more of the NOS criteria as high quality.

### Statistical analyses

RR with its 95%CI was used as a common measure of the association between obesity and risk of hip fracture across studies. Homogeneity of RRs across studies was tested by the I^2^ statistic, which is a quantitative measure of inconsistency across studies [32]. The pooled RR were computed using either fixed-effects models [33] or, in the presence of heterogeneity (I^2^ >50.0%), random-effects models [34]. To find the possible heterogeneity, we performed subgroup analyses by gender. We also used meta-regression to investigate if there is a difference in effect between men and women. Because characteristics of populations, and adjustments for confounding factors were not consistent between studies, we further conducted a sensitivity analysis to explore possible explanations for heterogeneity and to examine the influence of various exclusion criteria on the overall pooled RR. We investigated the influence of a single study on the overall risk estimate by omitting 1 study in each turn. Potential publication bias was assessed by visual inspection of the Begg's funnel plots in which the logRRs was plotted against their stand errors (SEs) [35]. We also performed the Begg's rank correlation test and Egger's linear regression test to assess the publication bia [35], [36]. All analyses were performed using STATA version 11.0 (StataCorp LP, College Station, Texas). A P value <0.05 was considered statistically significant, except where otherwise specified.

## Results

### Study characteristics

**Figure 1:**
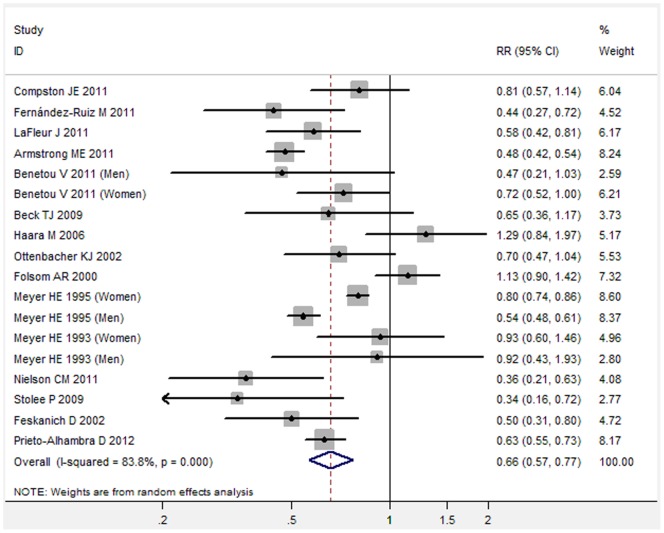
Forest plot showed an association between adult obesity and decreased risk of hip fracture (Meta-analysis of total included studies).

We initially retrieved 1326 unique citations from the PubMed and Embase databases. Of these, the majority were excluded after the first screening based on abstracts or titles, mainly because they were reviews, case-control studies, cross-sectional studies, or not relevant to our analysis. 23 full-text papers were preliminarily included into this study. After full-text review of those papers, 5 studies were excluded for lack of necessary dada and 3 studies were excluded because they used a retrospective cohort design. Finally, 15 studies with a total of 3,126,313 participants were included in our meta-analysis (Supply figure 1) [15]–[29]. The characteristics of the 15 prospective cohort studies are presented in Table 1. These studies were published between 1993 and 2011 [15]–[29]. Seven studies were conducted in the United States [17], [18], [22], [24], [25], [27], [29], 7 in Europe [15], [16], [19]–[21], [23], [26], and 1 in Canada [28]. The mean length of follow-up ranged from 1 to 16.4 years. Three studies reported the RRs by gender and the data were extracted as RR for men and RR for women, respectively. The sizes of the cohorts ranged from 3050 to 9,006 (total 3,126,313). Outcome assessments were from a variety of sources, including medical record, self-report, and hospital database. Seven studies reported adjusted RRs, and the mainly including age, diabetes, cholesterol, and smoking. According to the NOS system, thirteen studies were considered as high quality (meeting 5 or more of the NOS criteria) [16]–[19], [21]–[26], [28], [29].

**Table 1:** Characteristics of 15 prospective cohort studies included into this meta-analysis.

study	Enrolment time	Setting	Number of participants	Definition	Follow up	Adjustment
Meyer HE 1993 [16]	1974–1978	Norway	52313	BMI ≥28.0 kg/m2	10 years	Age, height, physical activity, diabetes mellitus, and smoking
Meyer HE 1995 [19]	1963–1975	Norway	673848	Women BMI >29.3 kg/m2; Men BMI >27.5 kg/m2	16.4 years	None
Folsom AR 2000 [18]	1985–1986	USA	31702	BMI ≥30.2 kg/m2	11 to 12 years	Age, educational level, physical activity, alcohol intake, smoking status, vitamin use, et al.
Ottenbacher KJ 2002 [27]	1998–1999	USA	3050	BMI ≥30.0 kg/m2	7 years	None
Feskanich D 2002 [29]	1976	USA	61200	BMI ≥30.0 kg/m2	12 years	Age, smoking, postmenopausal hormone use, and intakes of calcium, vitamin use, et al.
Haara M 2005 [15]	1978–1980	Finland	3561	BMI ≥30.0 kg/m2	15 years	None
Beck TJ 2009 [24]	1991–2002	USA	4642	BMI ≥30.0 kg/m2	8.5 years	None
Stolee P 2009 [28]	2002–2006	Canada	40279	BMI ≥35.0 kg/m2	5 years	Age, smoking, height, et al.
Benetou V 2011 [20]	1992–2000	Europe	27982	BMI ≥30.0 kg/m2	8 years	Age at recruitment, educational level, smoking habits and history of diabetes mellitus at enrolment.
Armstrong ME 2011 [23]	1996–2001	UK	925345	BMI ≥30.0 kg/m2	6.2 years	None
LaFleur J 2011 [22]	1995–2005	USA	190847	BMI ≥30.0 kg/m2	29.1 months	None
Fernandez-Ruiz M 2011 [21]	1994–1995	Spain	5278	BMI ≥30.0 kg/m2	13 years	Age, gender, osteoporosis, circulatory disorders, et al.
Compston JE 2011 [17]	2006–2008	USA	60393	BMI ≥30.0 kg/m2	2 years	None
Nielson CM 2011 [25]	2000–2002	USA	5995	BMI ≥30.0 kg/m2	7 years	None
Prieto-Alhambra D 2012 [26]	2009	Spain	1,039,878	(BMI≥30 kg/m2	1 year	Age, smoking status, high alcohol intake, type 2 diabetes, and oral corticosteroid use.

(BMI, body mass index).

### Adult obesity and risk of hip fracture

Overall, there was substantial heterogeneity observed among those studies (I^2^ = 83.8%). Figure 1 showed the results from the random-effects model combining the RRs for hip fracture (Figure 1). Individuals with obesity compared with the reference group had a significantly decreased risk for developing hip fracture (RR: 0.66, 95% CI 0.57 to 0.77, P<0.001) (Figure 1). Further exclusion of any single study did not materially alter the overall combined RR.

**Figure 2:**
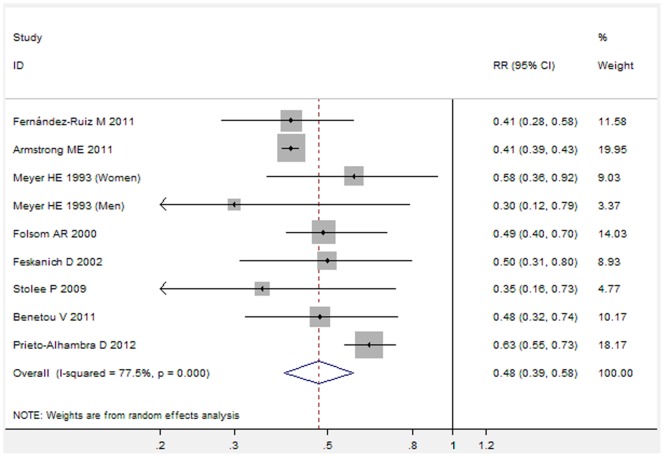
Forest plot showed an association between adult obesity and decreased risk of hip fracture (Analysis of adjusted RRs).

**Figure 3:**
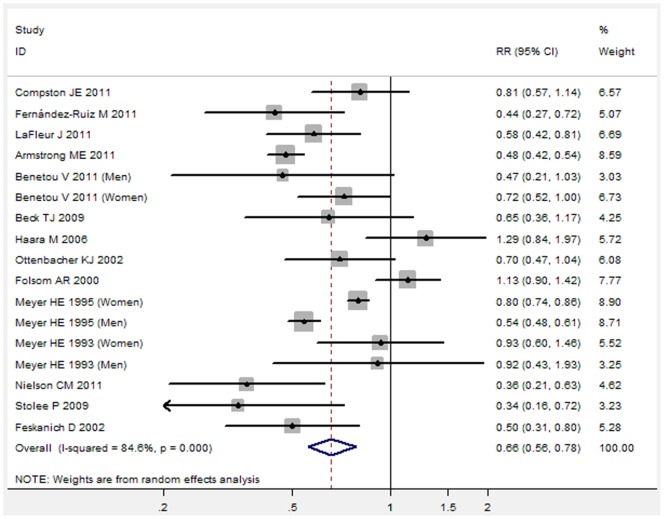
Forest plot showed an association between adult obesity and decreased risk of hip fracture (Analysis of unadjusted RRs).

Subgroup analyses were firstly performed by the adjusted RRs or unadjusted RRs. Figure 2 showed the results from the random-effects model combining the adjusted RRs for hip fracture (Figure 2). Individuals with obesity compared with the reference group had a significantly decreased risk for developing hip fracture (adjusted RR: 0.48, 95% CI 0.39 to 0.58, P<0.001). Further exclusion of any single study also did not materially alter the overall combined RR. Figure 3 showed the results from the random-effects model combining the unadjusted RRs for hip fracture (Figure 3). Individuals with obesity compared with the reference group had a significantly decreased risk for developing hip fracture (unadjusted RR: 0.66, 95% CI 0.56 to 0.78, P<0.001). Further exclusion of any single study also did not materially alter the overall combined RR.

Subgroup analyses were then performed by gender. Individuals with obesity compared with the reference group had a significantly decreased risk for developing hip fracture in both men (RR 0.54, 95% CI 0.48 to 0.60, P<0.001) and women (RR 0.70, 95% CI 0.58 to 0.84, P<0.001). Further exclusion of any single study did not materially alter the overall pooled RRs above. Besides, meta-regression was performed to investigate if there was a difference in effect between men and women, but we found that there was no obvious difference in effect between men and women (P>0.05).

### Risk of publication bias

**Figure 4:**
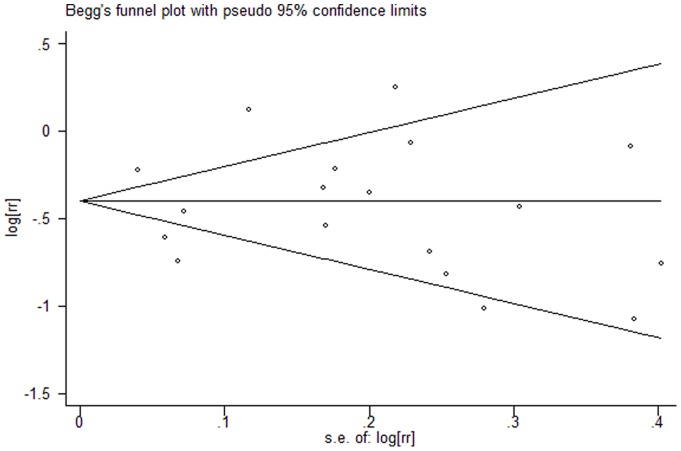
Begg's funnel plot did not identify substantial asymmetry in the meta-analysis of total 15 studies.

Visual inspection of the Begg funnel plot did not identify substantial asymmetry (Figure 4). The Begg rank correlation test and Egger's linear regression test also indicated no evidence of publication bias (Begg's rank correlation test, P = 0.89; Egger's linear regression test, P = 0.73). Besides there was also no evidence of publication bias in the subgroup analyses (Table 2). Thus, there was no obvious risk of publication bias in this meta-analysis.

**Table 2:** Summary of the results in the meta-analysis of adult obesity and risk of hip fracture.

Outcomes	No of studies	RR(95% CI)	P value	Pooled model	I^2^	P _Egger test_
**Total population**						
Total studies	18	0.66(0.57–0.77)	<0.001	Random	83.8%	0.732
Unadjusted RR	17	0.66(0.56–0.78)	<0.001	Random	84.6%	0.726
Adjusted RR	9	0.48(0.39–0.58)	<0.001	Random	77.5%	0.364
**Women**						
Total studies	10	0.70(0.58–0.84)	<0.001	Random	86.6%	0.845
Unadjusted RR	9	0.71(0.57–0.89)	<0.001	Random	87.6%	0.851
Adjusted RR	5	0.51(0.40–0.66)	<0.001	Random	88.4%	0.256
**Men**						
Total studies	4	0.54(0.48–0.60)	<0.001	Fixed	26.9%	0.912
Unadjusted RR	4	0.54(0.48–0.60)	<0.001	Fixed	26.9%	0.912
Adjusted RR	1	0.30(0.12–0.77	0.012	Fixed	NA	NA

(NA = not applicable).

## Discussion

There is rapidly growing interest in the association between obesity in adults and risk of hip fracture. Many observational studies have been published to evaluate the association between obesity in adults and risk of hip fracture, but controversial results are reported. To comprehensively evaluate the association between obesity in adults and risk of hip fracture, we conducted a meta-analysis of 15 prospective cohort studies with a total 3,126,313 participants [15]–[29]. Our meta-analysis provides evidence that obesity in adults is significantly and independently associated with a decreased risk of hip fracture. Adults with obesity compared with the reference group had a significantly decreased risk for developing hip fracture (RR: 0.66, 95% CI 0.57 to 0.77, P<0.001; adjusted RR: 0.48, 95% CI 0.39 to 0.58, P<0.001; unadjusted RR: 0.66, 95% CI 0.56 to 0.78, P<0.001). Subgroup analyses by gender suggest with obesity compared with the reference group have a significantly decreased risk for developing hip fracture in both men (RR 0.54, 95%CI 0.48 to 0.60, P<0.001) and women (RR 0.70, 95% CI 0.58 to 0.84, P<0.001). Thus, our meta-analysis suggests that obesity in adults significantly decreases the risk of hip fracture, and obesity is probably a protective factor of hip fracture in adults.

Low bone mineral density (BMD) has been considered one of the major causes of the increased risk of fracture in those with low weight, while higher BMD in adults is posited to mitigate risk of fractures [10], [37]. Body weight is directly associated with BMD, and a low BMI has been identified as an important risk factor for lower BMD and predicts greater bone loss in older age [13]. On the other hand, BMD is moderately positively correlated with BMI, many obese individuals have relatively higher BMD, and bone strength may increase in proportion to increases in total or fat mass. An increased strain on the bones imposed by higher body mass may lead to increased BMD and improved structural integrity of the bones. The higher BMD and bone mineral content in obesity are attributed to multiple factors. These include a greater mechanical loading on bone and the altered hormonal milieu and higher serum levels of adipokines associated with obesity, which play an important role in influencing bone mass in obesity [13]. Thus, obesity is an important protective factor for fractures by an effect mediated predominantly through high BMD [13]. Besides, obesity is also widely believed to be protective against fracture because of the effect of increased soft-tissue padding. Individuals who carry more fat mass may benefit from cushioning of their hip by gluteofemoral adipose tissue, which reduces impact forces when they fall and hence their chance of fracture [13].

To our knowledge, this is the first comprehensive systematic review and meta-analysis on the effect of obesity on the risk of hip fracture in adults. We carried out an extensive quality assessment, and investigated heterogeneity with sensitivity analyses and subgroup analyses. We compared the results of crude and adjusted data to try to determine if the observed risks were due to body mass index independently or were explained by confounding factors. We also performed subgroup analyses by gender, to explore if the gender specific effect existed. All those above could add the strength of evidence for the protective effect obesity on hip fracture in adults from our meta-analysis. Besides, we conclude that obesity is a protective factor of hip fracture in adults and it is similar in both sexes.

Obesity is growing concerns worldwide, and an estimated 1.5 billion people were obese in the year 2008 worldwide, and 34% of the population of the United States is obese [13], [38], [39]. Obesity has led to an increased risk for several comorbidities, including cardiovascular disease (CVD), diabetes, and certain cancers [40]–[42]. In addition, although weight reduction is recommended to reduce comorbidities related to obesity, it also induces bone loss and increases risk of fracture in older individuals [13], [14]. Besides, a high BMD can be due to increased physical activity or obesity, and bone loss could result in an increased risk of fractures due to weight reduction [13]. Since obesity is a protective factor of hip fracture in adults and it is similar in both sexes, dietary and clinical interventions can be used to help attenuate bone loss with weight reduction during weight reduction, such as increasing a specific micro- or macronutrient and physical activity [13].

Limitations of this systematic review include the obvious inconsistency across studies, especially in the confounding factors and lengths of follow up. Some confounding factors might account for the observed association between obesity and risk of hip fracture, which were not adjusted for in some of those included studies. Among those studies adjusted for confounding factors, those factors varied greatly among different studies. Besides, the length of follow up also varied greatly among different studies, arranging from 1 to 16.4 years. In addition, we did not try to contact the authors of those included studies for relevant information where it was not available in published form, and there was also possibility of having missed studies in the literature search and selective presenting of results from those included studies, therefore a meta-analysis of individual patients data is urgently needed to further provide a more precise estimation on the effect of obesity on hip fracture. Finally, we pooled data based on the original studies' definitions of obesity. There was a problem of varying cut-offs of definitions of obesity between studies. All the inconsistency across studies could cause the obvious heterogeneity in this meta-analysis (Table 2).

In conclusion, this meta-analysis of prospective cohort studies suggests that obesity in adults significantly decreases the risk of hip fracture, and obesity is probably a protective factor of hip fracture in adults.

## Supporting Information

Figure S1 Flow Diagram in this meta-analysis.
(DOC)

Table S1 PRISMA 2009 Checklist in this meta-analysis.
(DOC)
