# Supplementary material for: Obesity and Risk of Hip Fracture in Adults: A Meta-Analysis of Prospective Cohort Studies
Source: PLoS One. 2013 Apr 12;8(4):e55077. doi: 10.1371/journal.pone.0055077 (PMC3625172; doi:10.1371/journal.pone.0055077)
Supplement: Figure S1 — Flow Diagram in this meta-analysis. (DOC) [file pone.0055077.s001.doc]

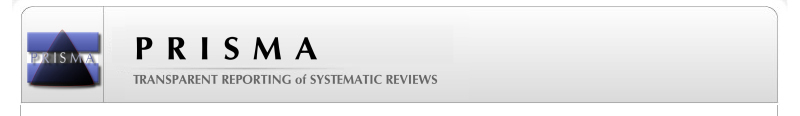
**PRISMA 2009 Flow Diagram**

**Screening**

**Included**

**Eligibility**

**Identification**

Records identified through database searching
(n = 1321 )

Additional records identified through other sources
(n = 5 )

Records after duplicates removed
(n = 1326 )

Records screened
(n = 1326 )

Records excluded
(n = 1303 )

Full-text articles assessed for eligibility
(n = 23)

Full-text articles excluded, with reasons
(n = 8 )

5 for lack of useful data;

3 for retrospective cohort design

Studies included in qualitative synthesis
(n =15 )

Studies included in quantitative synthesis (meta-analysis)
(n = 15 )
